# Nickel–Iron-Layered Double Hydroxide Electrocatalyst with Nanosheets Array for High Performance of Water Splitting

**DOI:** 10.3390/molecules29092092

**Published:** 2024-05-01

**Authors:** Zhi Lu, Shilin Li, Laiyuan Ning, Kun Tang, Yifan Guo, Long You, Chong Chen, Guangxin Wang

**Affiliations:** 1 School of Materials Science and Engineering, Henan University of Science and Technology, Luoyang 471003, China; 2 Henan Engineering Research Center for High Purity Materials and Sputtering Targets, Luoyang 471003, China; 3 School of Material Science and Engineering, Zhengzhou University, Zhengzhou 450001, China

**Keywords:** electrocatalysis, Ni-based, layered double hydroxides, hydrolysis of water

## Abstract

Developing high-performance and cost-competitive electrocatalysts have great significance for the massive commercial production of water-splitting hydrogen. Ni-based electrocatalysts display tremendous potential for electrocatalytic water splitting. Herein, we synthesize a novel NiFe-layered double hydroxide (LDH) electrocatalyst in nanosheets array on high-purity Ni foam. By adjusting the Ni/Fe ratio, the microstructure, and even the behavior of the electrocatalyst in the oxygen evolution reaction (OER), changes significantly. The as-obtained material shows a small overpotential of 223 mV at 10 mAcm^−2^ as well as a low Tafel slope of 48.9 mV·dec^−1^ in the 1 M KOH electrolyte. In addition, it can deliver good stability for at least 24 h of continuous working at 10 mAcm^−2^. This work proposes a strategy for engineering catalysts and provides a method for the development of other Ni-based catalysts with excellent performance.

## 1. Introduction

With the advantage of a high heat value and convenience for both use and transport, H_2_ energy has been known as an environmentally friendly and splendid energy carrier, and a promising energy source to replace fossil fuels [1]. Separation of H_2_ from the air is uneconomical because its proportion in the air is too low, so most of the H_2_ used in the industry comes from the dissociation of fossil fuels. This causes not only an enormous waste of resources, but also environmental pollution and enormous greenhouse gas emissions [2]. Meanwhile, the global demand for hydrogen has risen from 59 million tons in 2000 to 74 million tons in 2010 and then to 88 million tons in 2020. It is predicted that the demand will rise to 211 million tons in 2030 and 528 million tons in 2050. Before 2020, hydrogen was mainly used in industrial processes. In future, it will be widely used in transport, NH_3_ fuel, synfuels, buildings, etc. It is estimated that the hydrogen-related industrial chain will bring about USD 7–8 trillion worth of investments by 2050 and create USD 3 trillion worth of revenues [3].

Among the approaches to produce H_2_, water splitting is the most sustainable and environmentally friendly means [4,5,6]. As the devices for water electrolysis are simple and easy to control, its industrialization is potential and profitable. Water splitting involves the HER and the OER, in which the OER is always a restricted process, so it is a key factor in water electrolysis [7,8]. To improve the efficiency of water splitting, many technologies have been developed to reduce the OER overpotential [9,10,11]. Nowadays, some precious metal-based electrocatalysts, for example, Pt-based and Ir-based electrocatalysts, seem most effective in HERs and OERs, which may be due to their electronic orbit structure, which can decrease the overpotential and increase the rate of electrolysis reaction significantly [12,13]. But the small reserve and costs hindered their wide application. 

Recently, many efforts were put into developing low-cost metal-based electrocatalysts. Among them, transition metal-layered double hydroxides (LDHs) have showed exceptional catalytic behavior and stability when used in water splitting [14,15,16]. LDHs are easy to operate and provide sufficient surface active sites through an intermediate when undergoing polarization during the reaction [17,18,19]. This attributes to their unique microstructure and is a benefit to their efficiency of water splitting. There is plenty of research about LDHs, for example, the Ni–Al-based LDH [20,21], Ni–Co-based LDH [22,23], Co–Zn-based LDH [15,24], etc. For example, Feng et al. synthesized a nano-porous NiAl-LDH based on nickel foam via the hydrothermal process [20]. The unique microstructure endowed the material with an optimized electron orbit structure and sufficient active sites, which would contribute to the electricity and mass conductivity and mass transportation. The Ni-Al-based LDH showed an excellent property in water splitting. Zhang et al. reported a ZnFe-LDH OER cocatalyst that integrated with anatase and rutile TiO_2_ via the photoassisted electrodeposition method [25]. By virtue of coordination, O vacancies were introduced into the composite electrode and contributed to the further regulation of isolation and transfer of the charge carriers, which could improve the separation efficiency of the carriers. The existence of the ZnFe-LDH can ensure the accelerated kinetics of the water oxidation reaction in the electrode surface. Then, an excellent catalytic activity in water splitting would be obtained. Wang et al. prepared a MnFeCoNiCu LDH via the hydrothermal method and decorated it with Au atoms and O vacancies through electrochemical deposition [26]. It was found that the decoration of single atoms contribute to upshifting O atom orbits and weakening the metal–O bonds, which would be conducive to activate and redox the related lattice oxygen. Then, the multielement LDH showed excellent OER activity and stability. The OER behavior depends on the unique property of the as-prepared composite, as well as its connection to the morphology [27,28]. The nanostructure catalysts possess quite a stable structure and high surface area to provide sufficient active sites; a favorable electron and mass transfer capacity will be presented to them, thereby allowing a considerable enhanced electrocatalytic performance to be achieved [29,30].

Herein, we report a series of NiFe-LDH nanosheets array on high-purity nickel foam by a one-step hydrothermal process. Hydrothermal processes are inexpensive and benefit regulation of the microstructure and further affect the material performance. The microstructure of the composite can be adjusted through adjusting the Ni/Fe ratio. The nanosheets array can provide sufficient active sites during the hydrolysis reaction. Excellent OER performance and stability have been obtained. This work proposes a strategy for engineering catalysts and provides inspiration for the development of other Ni-based catalysts with excellent performance.

## 2. Results and Discussion

### 2.1. Characterization of the Electrocatalyst

**Figure 1:**
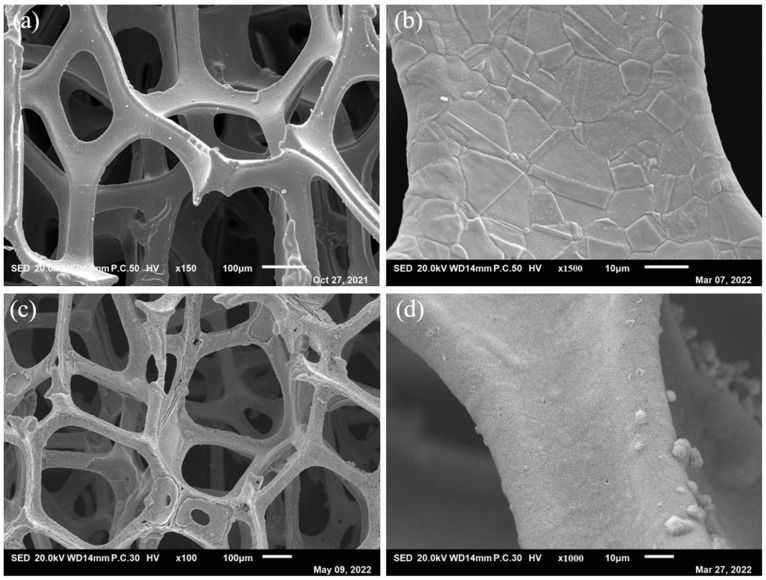
SEM images of (**a**,**b**) treated NF and (**c**,**d**) NiFe_3_-LDH/NF after preparation.

Through hydrothermal processes, the Ni–Fe-based LDH nanosheets grew on the NF substrate. Comparing the SEM images of the NF surfaces before and after preparation shows that the NF obviously becomes rough, as shown in Figure 1, which are the samples on the NF surface. The “treated NF” in Figure 1 means the new Ni foam was cleaned by HCl solution, ethanol and ultrapure water. 

**Figure 2:**
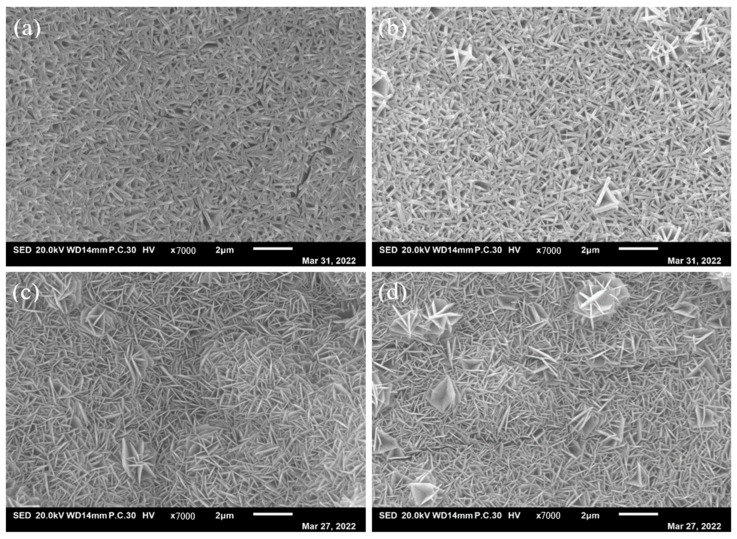
Low magnification morphologies of (**a**) NiFe-LDH, (**b**) NiFe_2_-LDH, (**c**) NiFe_3_-LDH, and (**d**) NiFe_4_-LDH nanosheets array.

**Figure 3:**
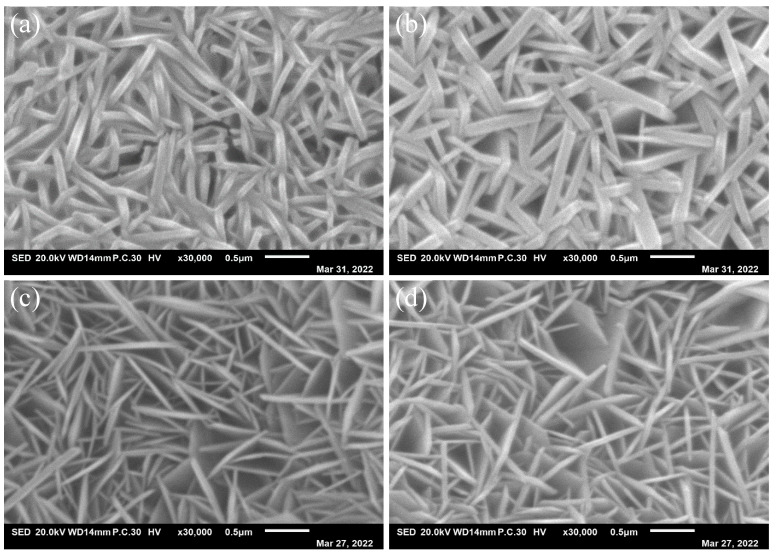
High magnification morphologies of (**a**) NiFe-LDH, (**b**) NiFe_2_-LDH, (**c**) NiFe_3_-LDH, and (**d**) NiFe_4_-LDH nanosheets array.

To further explore the influence of synthesis, low magnification morphologies, high magnification morphologies and XRD of the Ni–Fe-based LDH have been carried out. As shown in Figure 2, the as-prepared electrocatalysts show a morphology of nanosheets. But the microstructure of the electrocatalysts have a significant difference from the others. Figure 3 is the high magnification morphologies of the as-prepared electrocatalyst. As can be seen, when the molar ratio of Ni:Fe was 1:1, the microstructure of the Ni–Fe LDH showed thick nanosheets and the nanosheets were distributed densely. When the molar ratio was 1:2, it seemed that the nanosheets became thick and the nanosheets were still distributed very densely, which may be no good for the catalytic performance. If the Fe element was increased continually, significant changes would be observed. When the molar ratio was adjusted to 1:3 and 1:4, the nanosheets became thinner obviously. The number of pores between the nanosheets increased greatly, which is good for the catalytic performance. The average length and average thickness of the nanosheets can be measured easily. 

Table 1 is the parameters of the specimens in Figure 3. In the table, “Length” is the average value of the length of the nanosheets; “Thickness” is the average value of the thickness of the nanosheets, “Ratio of L/T” is the ratio of “Length” to “Thickness”. As shown in Table 1, the lengths of NiFe-LDH/NF, NiFe_2_-LDH/NF, NiFe_3_-LDH/NF and NiFe_4_-LDH/NF are 0.72 μm, 1.083 μm, 0.934 μm and 0.846 μm, respectively. The thickness values of NiFe-LDH/NF, NiFe_2_-LDH/NF, NiFe_3_-LDH/NF and NiFe_4_-LDH/NF are 84 nm, 107 nm, 69 nm and 73 nm, respectively. Through calculation, it is found that the Ratio of L/T values of NiFe-LDH/NF, NiFe_2_-LDH/NF, NiFe_3_-LDH/NF and NiFe_4_-LDH/NF are 8.57, 10.12, 13.54, and 11.59. 

Parameters such as the thickness of the nanostructure may be affected by the ratio of metal cation in the metal-based LDHs, thereby affecting the performance of the electrocatalyst [31]. Combining the microstructure morphologies and parameters above, it can be concluded that with the increasing Fe element, the ratio of length/thickness increases and then decreases. To obtain a larger total surface area, the molar ratio of Ni:Fe should be kept as 1:3, which may contribute to increasing the active sites and promoting OERs.

**Figure 4:**
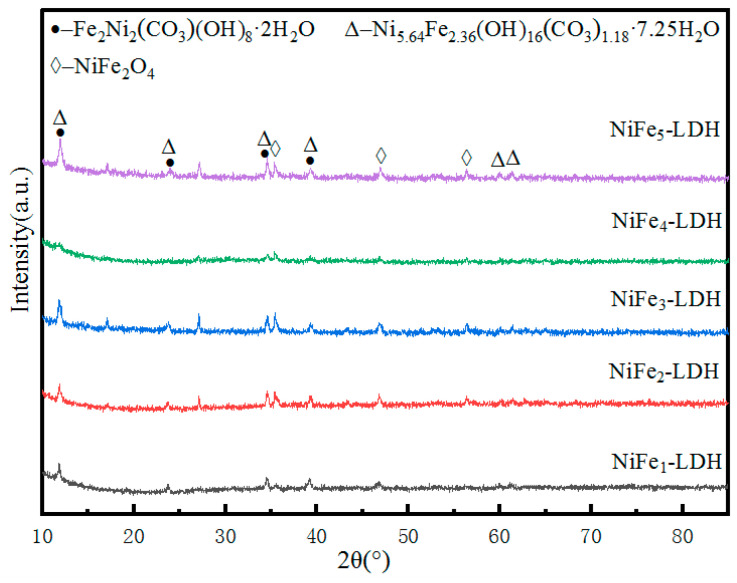
XRD patterns of NiFe-based LDH.

Figure 4 is an X-ray diffraction of the Ni–Fe-based LDH that excludes the effect of the Ni foam substrate. As shown, the diffraction peaks at 11.8°, 23.8°, 34.7°, and 39.3° correspond to the planes (003), (006), (012), and (015) of Fe_2_Ni_2_(CO_3_)(OH)_8_·2H_2_O (JCPDF, No. 49-0188) and Ni_5.64_Fe_2.36_(OH)_16_(CO_3_)_1.18_·7.52H_2_O (JCPDF, No. 51-0463). The diffraction peaks at 35.7°, 37.3°, and 56.3° correspond to the planes (311), (222), and (511) of NiFe_2_O_4_ (JCPDF, No. 10-0325). It indicates that the Ni–Fe-based LDH electrocatalyst with nanosheets array has been synthesized on the high-purity Ni foam successfully. 

**Figure 5:**
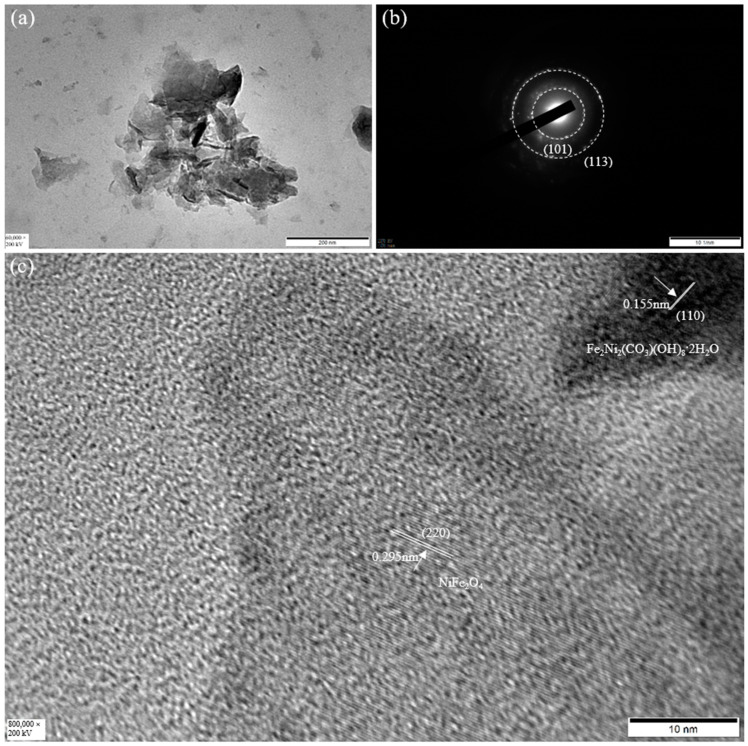
(**a**) TEM image of NiFe_3_-LDH, (**b**) SAED image of NiFe_3_-LDH, and (**c**) HRTEM image of NiFe_3_-LDH.

To deeply investigate the microstructure of the as-prepared electrocatalyst, Figure 5 shows a TEM analysis of the NiFe_3_-LDH. It shows that the NiFe_3_-LDH consists of ultrathin nanosheets, with dimensions between 100 and 200 nm (Figure 5a), which is beneficial for the exposure of surface active sites for the reaction. Figure 5b is a selected area electron diffraction; it shows two light diffraction rings, which correspond to the (101) plane of Fe_2_Ni_2_(CO_3_)(OH)_8_·2H_2_O and the (113) plane of Ni_5.64_Fe_2.36_(OH)_16_(CO_3_)_1.18_·7.52H_2_O, respectively. The interplanar distances of the lattice fringes in Figure 5c were 0.155 nm and 0.295 nm, corresponding to the (110) plane of Fe_2_Ni_2_(CO_3_)(OH)_8_·2H_2_O and the (220) plane of NiFe_2_O_4_ [32], respectively.

**Figure 6:**
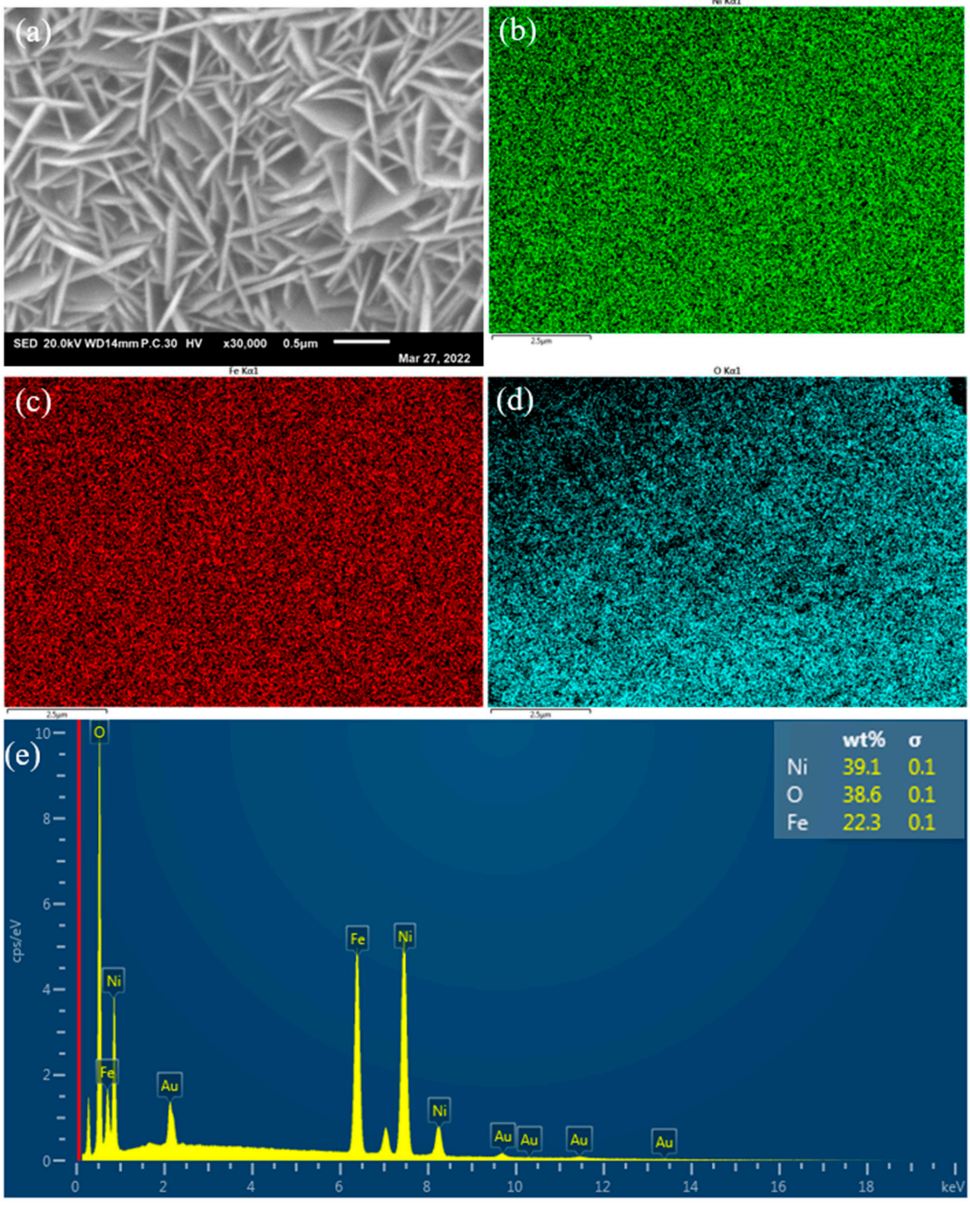
EDS of NiFe_3_-LDH nanosheets array. (**a**) SEM image of NiFe_3_-LDH, the distribution of (**b**) Ni, (**c**) Fe, (**d**) O, (**e**) Surface energy spectrum of NiFe_3_-LDH.

Moreover, the EDS images (Figure 6) verified that Ni, Fe, and O have been distributed uniformly throughout the entire surface of the sample. The results may induce greater stability during application of the electrocatalysts. The EDS data in Figure 6e shows Ni 39.1 wt% and Fe 22.3 wt%. According to the international atomic weight of Ni and Fe, the atom ratio of Ni/Fe can be worked out to be 1.75, which lies inbetween the values for Ni_5.64_Fe_2.36_(OH)_16_(CO_3_)_1.18_·7.52H_2_O and Fe_2_Ni_2_(CO_3_)(OH)_8_·2H_2_O. 

### 2.2. Electronic States of NiFe_3_-LDH

**Figure 7:**
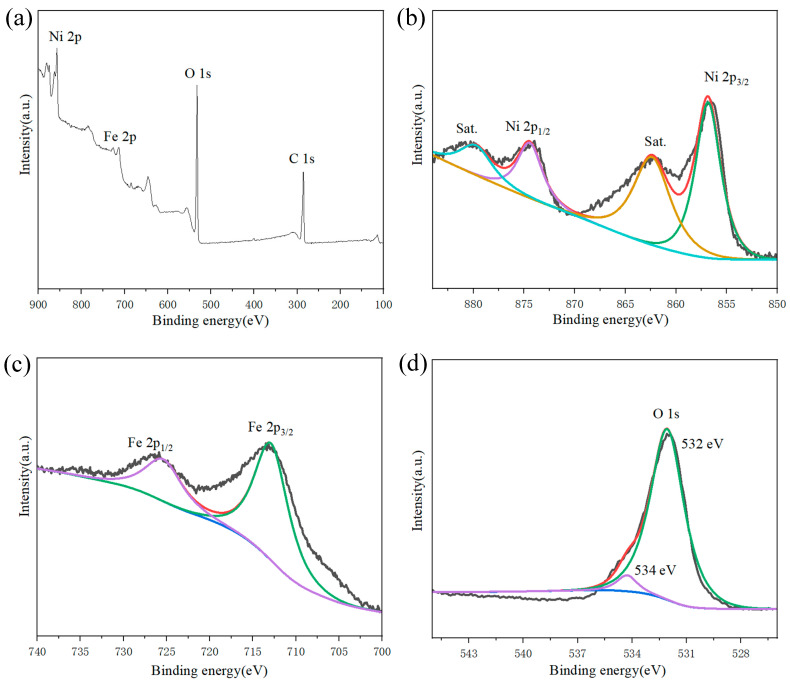
XPS spectra of (**a**) survey spectrum, (**b**) Ni 2p, (**c**) Fe 2p, and (**d**) O 1s in the NiFe_3_-LDH electrocatalyst.

The valent state of constituent atoms of the NiFe_3_-LDH were examined by XPS analysis. The XPS survey spectrum of the NiFe_3_-LDH (Figure 7a) indicates the four major elements, Ni, C, Fe, O, with the atomic percentages analyzed being C 48.4%, O 36.7%, Fe 3.8% and Ni 8.9%, respectively. The spectra of Ni 2p, C 1s, Fe 2p, and O 1s can be identified clearly.

The Ni 2p spectrum can be separated into Ni 2p_3/2_ and Ni 2p_1/2_ (Figure 7b), which are located at 856.6 eV and 873.9 eV along with the corresponding satellite peaks at 862.2 eV and 880.8 eV. These results are attributed to the presence of Ni^2+^ [33]. The Fe 2p spectrum (Figure 7c) can be separated into Fe 2p_3/2_ and Fe 2p_1/2_, which are located at 713.48 eV and 726.88 eV. Based on the XPS data, the atomic molar ratio of Ni:Fe:O = 2.38:1:9 is close to the stoichiometry of Ni_5.64_Fe_2.36_(OH)_16_(CO_3_)_1.18_, and this agrees with the formation of Ni_5.64_Fe_2.36_(OH)_16_(CO_3_)_1.18_·7.52H_2_O that was detected by the tests above [34].

### 2.3. Electrochemical Performance of NiFe-Based LDH

**Figure 8:**
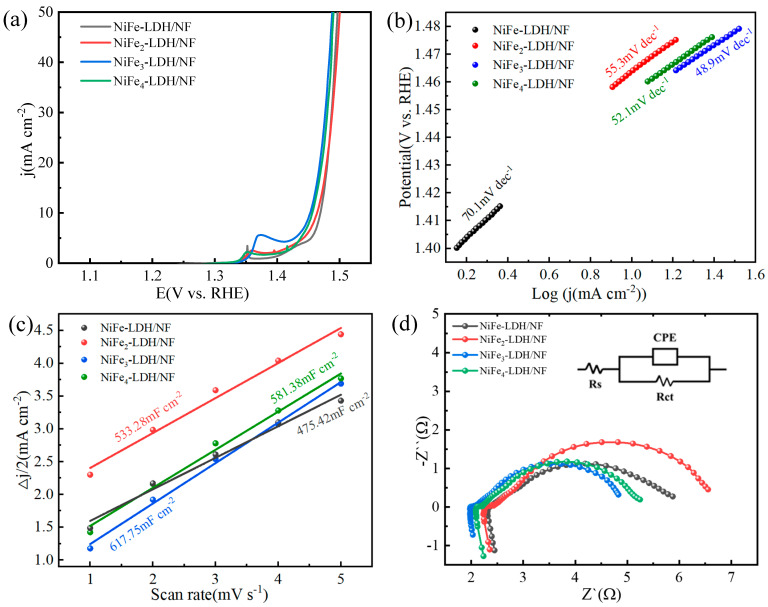
(**a**) LSV curves of NiFe−LDH, NiFe_2_−LDH, NiFe_3_−LDH, and NiFe_4_−LDH, (**b**) Tafel slopes of NiFe−LDH, NiFe_2_−LDH, NiFe_3_−LDH, and NiFe_4_−LDH, (**c**) Cdl curves of NiFe−LDH, NiFe_2_−LDH, NiFe_3_−LDH, and NiFe_4_−LDH, and (**d**) EIS tests of NiFe−LDH, NiFe_2_−LDH, NiFe_3_−LDH, and NiFe_4_−LDH. The inset diagram is the equivalent circuit.

The performance of the OER of the NiFe-based LDH was tested in the electrolyte of 1 M KOH at a 10 mA·cm^−2^ current density. The overpotential is subtracted from the potential value by 1.23 V. The NiFe_3_-LDH/NF shows the lowest overpotential of 223 mV (Figure 8a), which obviously has an advantage over NiFe-LDH (240 mV), NiFe2-LDH (234 mV), and NiFe_4_-LDH (227 mV).

The Tafel slopes of the NiFe-LDH/NF (70.1 mV·dec^−1^), NiFe_2_-LDH/NF (55.3 mV·dec^−1^), NiFe_3_-LDH/NF (48.9 mV·dec^−1^), and NiFe_4_-LDH/NF (52.1 mV·dec^−1^) are displayed in Figure 8b. The Tafel slope of the NiFe_3_-LDH/NF is smaller than that of the NiFe-LDH/NF, NiFe_2_-LDH/NF and NiFe_4_-LDH/NF, indicating the fast OER kinetics of the NiFe_3_-LDH/NF [35]. Taking the overpotential and Tafel slope data of the NiFe-LDH/NF, NiFe_2_-LDH/NF, NiFe_3_-LDH/NF and NiFe_4_-LDH/NF together, it indicates that the OER activity of the NiFe_3_-LDH/NF is far greater than the others. 

The oxygen evolution reaction of electrocatalysts in hydrolysis is deemed to be a four-step reaction. First, the molecules of water are adsorbed to the active sites of the nanostructure and are transformed into some kind of [OH] (the square brackets indicate an O vacancy on the surface) intermediate. Second, the intermediate is oxidized into [O] on the active sites. Third, [O] combines with the water molecule to form a [OOH] intermediate. Finally, the [OOH] breaks down into O_2_ [36]. It can be concluded that the performance of the electrocatalyst depends on the quantity of the active sites and the reaction kinetics between H_2_O and the intermediates. 

The electrochemical active surface area (ECSA) is always used to estimate the quantity of active sites in a catalytic reaction. It can be calculated from the following equation [37]: ECSA = C_dl_/C_s_, where C_dl_ and C_s_ are the electrochemical double-layer capacitance and the specific capacitance of the electrocatalyst. For the Ni foam-based electrocatalyst, C_s_ = 0.04 mF cm^−2^ [38]. 

The C_dl_ of the NiFe_3_-LDH/NF (61.75 mF·cm^−2^) is much higher than that of the NiFe-LDH/NF (475.42 mF·cm^−2^), NiFe_2_-LDH/NF (533.28 mF·cm^−2^), and NiFe_4_-LDH/NF (581.38 mF·cm^−2^) (Figure 8c). This indicates that there are more active sites in the NiFe_3_-LDH/NF, and the sufficient active sites may be rooted from the introduction of a moderate amount of the Fe element [37]. 

EIS was employed to further investigate the catalytic reaction kinetics (Figure 8d). The NiFe_3_-LDH/NF has the smallest semicircle diameter when compared to the others. The Rct value of the NiFe_3_-LDH/NF is 3.16 Ω, which is smaller than that of the NiFe-LDH/NF (3.59 Ω), NiFe_2_-LDH/NF (4.73 Ω), and NiFe_4_-LDH/NF (3.19 Ω), which demonstrates the faster charge transfer process of the NiFe_3_-LDH/NF. This may contribute to the accelerated charge transfer between the interfaces of the electrode and electrolyte [39,40]. 

The effect of Fe on the OER performance of the NiFe-based LDH/NF may be attributed to several factors [41]. Firstly, with increasing Fe content, the electronic interaction between Ni and Fe becomes strong, which may modify the electronic structure of the NiFe-based LDH and would be conducive to activate and redox the related lattice oxygen; secondly, the moderate introduction of the Fe element can increase the ratio of “Length” to “Thickness”, which may contribute to increasing the electrochemical surface area and active sites of the LDHs for the catalytic reaction [42]; and thirdly, the moderate addition of the Fe element may influence the absorption of [OH] and [O], weakening the Gibbs free energy of the transformation from [OH] to [O], and bring down the overpotential of the NiFe-based LDH in the OER [42,43]. Some research indicates that the low oxidation state of Ni cations benefits the OER activity of the catalyst. Ni plays an active role and Fe plays a stable role in NiFe catalysts. The presence of Fe in NiFe-based catalysts has a function to inhibit the electrochemical oxidation of Ni, so the transformation of NiOOH from Ni(OH)_2_ was suppressed. Then, the average oxidation state of the nominally Ni^3+^ sites decrease with Fe incorporation. This results in higher OER activity [44].

**Figure 9:**
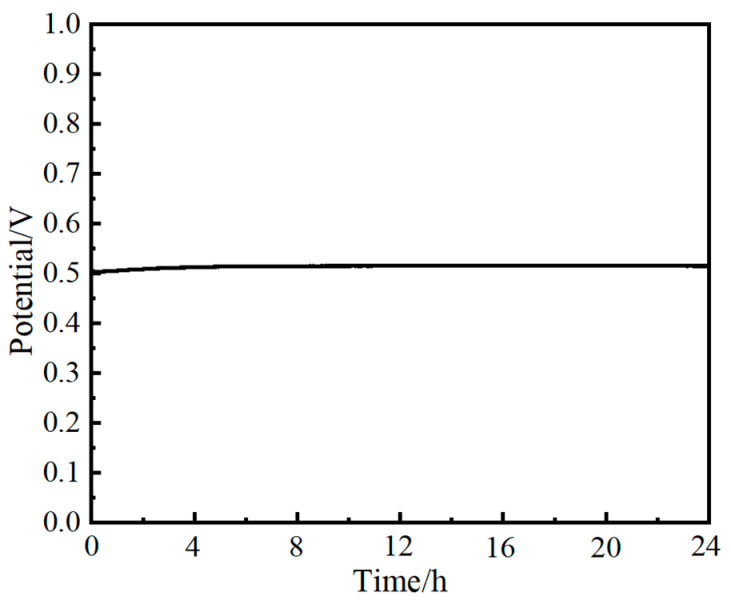
Stability curve of NiFe_3_-LDH/NF in chronoamperometry.

Stability of the electrocatalyst is a critical factor for application [45,46]. As shown in Figure 9, the NiFe_3_-LDH/NF exhibits quite a stable voltage under 10 mA·cm^−2^ after 24 h in 1.0 M KOH. The excellent stability is mainly attributed to the moderate doping of the Fe element.

## 3. Materials and Methods 

### 3.1. Materials

Fe (NO_3_)_3_·9H_2_O (99%), Ni (NO_3_)_2_·6H_2_O (99%), CO(NH_2_)_2_(99%), KOH (99%), ethyl alcohol (99%) and Ni foam (NF, 99.9%) were supplied by Aladdin (Shanghai, China). HCl, NH_4_F (99%) were purchased from Sinopharm Group (Beijing, China).

### 3.2. Electrocatalyst Preparation

Ni foams were tailored to a size of 2 cm × 2 cm and subjected to ultrasonic cleaning by 1 M HCl solution for 30 min. Then, they were cleaned by ethanol and ultrapure water several times to remove the impurities on the surface. Next, the samples were aired in a vacuum oven. NiFe-based LDH nanosheets will be prepared on Ni foams via a hydrothermal process. In a typical process, 1 mmol Ni(NO_3_)_2_·6H_2_O, 1 mmol CO(NH_2_)_2_, and 3 mmol Fe(NO_3_)_3_·9H_2_O into 50 mL was dissolved in ultrapure water and stirred continuously. Then, 10 mmol urea was added to the mixed solution. After stirring for 15 min, the cleaned Ni foams and the mixed solutions above were transferred into a 100 mL polyethylene reactor with a stainless-steel shell and kept at 160 °C for 5 h. When cooled to room temperature, the Ni foams are taken out and washed using ethanol and ultrapure water in turn, three times each, and then aired at 60 °C in a vacuum oven for one night. For comparison, several contents of Fe(NO_3_)_3_·9H_2_O (1 mmol, 2 mmol, 3 mmol, 4 mmol and 5 mmol) were used to synthesize the NiFe-based LDH, and the corresponding samples were flagged as the NiFe-LDH, NiFe_2_-LDH, NiFe_3_-LDH, NiFe_4_-LDH and NiFe_5_-LDH. 

### 3.3. Physical Characterization

XRD pattern was carried out by a X-ray diffractometer (Bruker-AXS D8 Advance) with Cu Kα radiation (5°/min, 10–85°). A field emission scanning electron microscope (JEOL JSM-7800F) was applied to examine the microscopic images and the energy dispersive spectroscopy of the electrocatalysts. A transmission electron microscopy (JEOL JEM-2100) was used to examine the TEM of the electrocatalysts. XPS was examined by a Thermo Scientific K-Alpha (Shanghai, China).

### 3.4. Electrochemical Performance

A standard CHI 660D electrochemical workstation (Tesco, Shanghai, China) was carried out to obtain the electrochemical behavior of the samples.

The obtained electrocatalysts were used as working electrodes, a Ag/AgCl electrode was applied as the reference electrode. Platinum was used as a counter electrode. The electrolyte was a 1 M KOH solution. Potentials in this work were converted into RHE following the equation E_RHE_ = E_Ag/AgCl_ + 0.1989 + 0.0591 × pH. The LSV curve was measured under 2 mVs^−1^ with iR compensation. Overpotentials were calculated following the equation η(V) = E_RHE_ − 1.23. EIS was measured from 0.01 to 10^4^ Hz. CV was measured at 1.0–5.0 mV/s. Electrochemical double-layer capacitance (C_dl_) was measured from CV. The stability was tested by chronoamperometry at 10 mA cm^−2^.

## 4. Conclusions

In summary, NiFe-based LDH/NF electrocatalysts with nanosheets array were successfully synthesized using a hydrothermal process. The ultrathin nanosheets microstructure of the NiFe-based LDH/NF can be regulated through controlling the Fe content. In comparison with the series of NiFe-based LDH/NF electrocatalysts, the NiFe_3_-LDH/NF exhibits outstanding OER behavior and stability. When using the NiFe_3_-LDH/NF as an OER catalyst, the overpotential is 223 mV at 10 mA·cm^−2^, and the Tafel slope is 48.9 mV·dec^−1^. These are significantly better than the others. The transformation of the ultrathin nanosheets microstructure may contribute towards increasing the catalytic active sites on the catalyst. This is conducive to improving the transfer of electrons between the electrocatalyst and electrolyte. In addition, NiFe-based LDH/NF electrocatalysts show excellent stability during reactions. This work can provide a new method to the producing of low-cost and efficient catalysts for water splitting.

## Figures and Tables

**Table 1:** Parameters of NiFe-based LDH.

Products	Length (µm)	Thickness (nm)	Ratio of L/T
NiFe-LDH	0.72	84	8.57
NiFe_2_-LDH	1.083	107	10.12
NiFe_3_-LDH	0.934	69	13.54
NiFe_4_-LDH	0.846	73	11.59

## Data Availability

The data presented in this study are available in the article.
